# Neuron-specific enolase has potential value as a biomarker for [^18^F]FDG/[^68^Ga]Ga-PSMA-11 PET mismatch findings in advanced mCRPC patients

**DOI:** 10.1186/s13550-020-00640-2

**Published:** 2020-05-24

**Authors:** Florian Rosar, Kalle Ribbat, Martin Ries, Johannes Linxweiler, Mark Bartholomä, Stephan Maus, Mathias Schreckenberger, Samer Ezziddin, Fadi Khreish

**Affiliations:** 1 Department of Nuclear Medicine, Saarland University—Medical Center, Kirrberger Str. 100, Geb. 50, 66421 Homburg, Germany; 2 Department of Urology, Saarland University—Medical Center, Homburg, Germany; 3 Department of Nuclear Medicine, University of Mainz, Mainz, Germany

**Keywords:** Prostate cancer, mCRPC, PSMA PET/CT, FDG PET/CT, Mismatch

## Abstract

**Background:**

PSMA-targeted radioligand therapy (PSMA-RLT) yielded impressive results in the metastasized castration-resistant prostate carcinoma (mCRPC) setting. High expression of PSMA is essential for successful PSMA-RLT. However, some patients develop [^18^F]FDG-avid lesions with low or no PSMA expression ([^18^F]FDG/[^68^Ga]Ga-PSMA-11 mismatch findings on PET/CT) in the course of treatment. Those lesions are not affected by PSMA-RLT and a change in therapy management is needed. To enable early mismatch detection, possible blood parameters as indicators for the occurrence of [^18^F]FDG/[^68^Ga]Ga-PSMA-11 mismatch findings on PET/CT were evaluated.

**Methods:**

Retrospective study of *N* = 66 advanced mCRPC patients with dual [^68^Ga]Ga-PSMA-11 and [^18^F]FDG PET/CT imaging within 4 weeks, who were referred for or received [^177^Lu]Lu-PSMA-617 radioligand therapy. Prostate-specific antigen (PSA), neuron-specific enolase (NSE), gamma-glutamyltransferase (GGT), and alkaline phosphatase (ALP) were tested as indicators for the occurrence of [^18^F]FDG/[^68^Ga]Ga-PSMA-11 mismatch findings. Additional to absolute values, relative changes (ΔPSA, ΔNSE, ΔGGT, ΔALP) over a period of 4 ± 1 weeks prior to [^18^F]FDG PET/CT were analyzed.

**Results:**

In total, 41/66 (62%) patients revealed at least one [^18^F]FDG/[^68^Ga]Ga-PSMA-11 mismatch finding on PET/CT. These mismatch findings were detected in 13/41 (32%) patients by screening for and in 28/41 (68%) patients during PSMA-RLT. NSE serum level (55.4 ± 44.6 μg/l vs*.* 18.5 ± 8 μg/l, *p* < 0.001) and ΔNSE (93.8 ± 124.5% vs*.* 2.9 ± 39.5%, *p* < 0.001) were significantly higher in the mismatch group than in the non-mismatch group. No significant differences were found for serum PSA (*p* = 0.424), ΔPSA (*p* = 0.417), serum ALP (*p* = 0.937), ΔALP (*p* = 0.611), serum GGT (*p* = 0.773), and ΔGGT (*p* = 0.971). For NSE and ΔNSE, the maximum value of the Youden index in ROC analysis was at a cut-off level of 26.8 μg/l (sensitivity 78%, specificity 96%) and at + 13.9% (sensitivity 84%, specificity 75%), respectively. An introduced scoring system of both parameters achieved a sensitivity of 90% and a specificity of 88% for the occurrence of [^18^F]FDG/[^68^Ga]Ga-PSMA-11 mismatch.

**Conclusion:**

We observed a significantly higher absolute serum concentration and a higher relative increase of NSE in advanced mCRPC patients with [^18^F]FDG-avid and insufficient PSMA expressing metastases ([^18^F]FDG/[^68^Ga]Ga-PSMA-11 mismatch findings on PET/CT) in our cohort. NSE might be used as a potential laboratory indicator for [^18^F]FDG/[^68^Ga]Ga-PSMA-11 mismatch findings, if this observation is confirmed in future, ideally prospective, studies in larger patient cohorts.

## Introduction

With over 1,000,000 new cases and approximately 300,000 deaths worldwide in 2012, prostate carcinoma is one of the most frequent malignant diseases in men [1]. A significant fraction of prostate carcinoma patients’ progresses to the lethal metastasized castration-resistant prostate carcinoma (mCRPC) setting [2, 3]. During the last decade, however, the development of new treatment options of men with mCRPC has led to an improved survival time [4]. In addition to chemotherapy with docetaxel or cabazitaxel [5, 6] and next-generation androgen receptor signaling inhibition with abiraterone or enzalutamide [7, 8], radioligand therapy targeting the prostate-specific membrane antigen (PSMA) is a potential option in palliative settings [9, 10]. PSMA-targeted radioligand therapy (PSMA-RLT) with [^177^Lu]Lu-PSMA-617 yielded impressive results in palliative settings while causing only moderate side effects [11, 12]. PSMA-targeted based positron emission tomography (PET)/computer tomography (CT) with radiolabeled PSMA ligands, such as [^68^Ga]Ga-PSMA-11, is frequently used for imaging of prostate cancer in clinical routine [13]. PSMA-targeted PET/CT is not only used for the staging of prostate cancer, but it is also a useful tool for therapy monitoring [14, 15] and indispensable for verifying PSMA expression prior to PSMA-RLT [16]. High expression of PSMA is essential for successful PSMA-RLT in patients with mCRPC. However, some patients develop lesions with low or no PSMA expression under ongoing treatment [17]. Those lesions are not affected by PSMA-RLT and a change in therapy management is needed [9, 18]. [^18^F]FDG PET/CT using ^18^F-labeled fluorodeoxyglucose ([^18^F]FDG) in addition to a PSMA-targeted PET/CT may be a suitable method for detection of those lesions. Mostly, prostate carcinoma cells have a low glucose metabolism due to energy gain by lipids and other energetic molecules but in advanced late-stage disease the glucose metabolism is highly increased by the Warburg effect and shifting to aerobic glycolysis after numerous mutation events [19]. Therefore, a combination of [^18^F]FDG and PSMA-targeted PET/CT may be able to detect clinically relevant glucose metabolic viable tumor lesions with low or no PSMA expression (mismatch lesions) in the advanced mCRPC setting. A recently prospective phase-II trial of [^177^Lu]Lu-PSMA-617 RLT excluded 16% of screened patients due to missing or low PSMA expression in [^18^F]FDG-avid metastases [20]. Early detection of these mismatch findings is thus needed to provide patients with alternative therapy options, additionally to or instead of PSMA-RLT [18]. However, frequently performing [^18^F]FDG PET/CT in addition to PSMA-targeted PET/CT is very cost intensive and associated with additional radiation exposure for the patient, which should be avoided even in a palliative setting.

In this study, selected serum parameters were therefore tested as indicators for the occurrence of [^18^F]FDG/[^68^Ga]Ga-PSMA-11 mismatch findings on PET/CT in this study, including the prostate-specific antigen (PSA) as the routine control and response parameter [21], alkaline phosphatase (ALP) as known to be elevated by bone metastases [22], gamma-glutamyltransferase (GGT) as a parameter of liver function affected by liver metastases [23], and neuron-specific enolase (NSE) as a possible parameter for transdifferentiating to a neuroendocrine type of prostate carcinoma [24].

## Materials and methods

### Study design

Retrospective monocenter study of mCRPC patients with dual [^68^Ga]Ga-PSMA-11 and [^18^F]FDG PET/CT imaging within 4 weeks, who were referred for or received [^177^Lu]Lu-PSMA-617 radioligand therapy at the clinic of nuclear medicine at Saarland University Medical Center from October 2015 till August 2019. Patients with secondary malignancies were excluded to avoid potential interference of image interpretation.

### Patients and ethics

*N* = 66 of in total 167 mCRPC patients referred for or received PSMA-RLT in our center were included in this retrospective study. Two of the 167 patients were excluded because of incomplete blood examination, 3/167 because of secondary malignancies and the remaining, and 96/167 due to missing or untimely [^18^F]FDG PET/CT. The patients received a [^68^Ga]Ga-PSMA-11 PET/CT and [^18^F]FDG PET/CT within a short time period prior to intended commencement of PSMA-RLT (*n* = 14/66) or in the course of PSMA-RLT (*n* = 52/66). The mean time between both PET/CT scans was 7.3 ± 10.7 days (95% confidence interval of the mean (CI) [4.6; 9.9]). The mean age of the patients was 69 years [range 45–89 years]. All patients received several pretreatments. Detailed information about the pretreatments and the patient characteristics is presented in Table 1. Androgen deprivation therapy (ADT) was continued unchanged in all patients to avoid variation of PSMA expression. [^68^Ga]Ga-PSMA-11 and [^18^F]FDG PET/CT were performed on a compassionate use basis under the German Pharmaceutical Act §13 (2b). Patients gave written consent after being thoroughly informed about the risks and potential side effects of this intervention. Additionally, patients consented to publication of any resulting data in accordance with the Declaration of Helsinki. Retrospective analysis approval was waived by the local institutional review board.

**Table 1:** Patient characteristics

Characteristic	Value
**Age [years]**	69 (45–89)
**PSA [ng/dl]**	70 (0.3–4742)
**Time from initial diagnosis [years]**	
≤ 2	18 (27.3%)
> 2 to ≤ 5	20 (30.3%)
> 5	28 (42.4%)
**Prior therapy**	
Prostatectomy	29 (44%)
Radiation	39 (59%)
ADT	66 (100%)
Enzalutamide	50 (76%)
Abiraterone	47 (71%)
Docetaxel	37 (56%)
Cabazitaxel	24 (36%)
Xofigo	7 (11%)
**ECOG PS before first cycle**	
0	18 (27%)
1	41 (62%)
2	5 (8%)
3	2 (3%)
**Site of metastasis**	
Bone	60 (91%)
Lymph node	49 (74%)
Liver	29 (44%)
Lung	10 (15%)

Data are presented as *n* (%) or median (range)

*Abbreviations*: *PSA* prostate-specific antigen, *ECOG PS* Eastern Cooperative Oncology Group Performance Status, *ADT* androgen deprivation therapy

### PET acquisition and analysis

For PET imaging, a mean activity of 124.1 ± 14.4 MBq [^68^Ga]Ga-PSMA-11 (CI [120.6; 127.6]) and 268.6 ± 28.7 MBq [^18^F]FDG (CI [261.6; 275.7]) was administered, followed by a 500-ml infusion of NaCl 0.9%. Fasting mean blood glucose value was 98.1 ± 17.3 mg/dl (CI [93.8; 102.4]) before administration of [^18^F]FDG. The mean uptake time was approximately 60 min (61.8 ± 6.6 min, CI [60.1; 63.4]) for [^68^Ga]Ga-PSMA-11 according to standard procedures for prostate cancer imaging [25] and 90 min (91.6 ± 8.7 min, CI [89.4; 93.7]) for [^18^F]FDG, according to the our standard procedure and German guideline for tumor imaging [26]. Before data acquisition, all patients were advised to empty their bladder. No diuretics were applied. All PET/CT scans were performed using a Biograph 40 mCT PET/CT scanner (Siemens Medical Solutions, Knoxville, TN, USA) with EANM Research Ltd. accreditation. The PET acquisition was performed from vertex to mid-femur with 3-min acquisition time per bed position. A bed position covers 21.4-cm extended field-of-view (TrueV). The PET datasets were reconstructed using an iterative 3-dimensional OSEM (ordered-subset expectation maximization) algorithm (3 iterations; 24 subsets) with Gaussian filtering and a slice thickness of 5 mm. Random correction, decay correction, scatter attenuation, and attenuation correction were applied. The CT was performed in low-dose technique using an X-ray tube voltage of 120 keV and a modulation of the tube current by applying CARE Dose4D with a maximal tube current-time product of 30 mAs. All PET/CT data sets were visually analyzed using certified analysis software (Sectra PACS—Sectra Medical Systems GmbH, Cologne, Germany). [^18^F]FDG and [^68^Ga]Ga-PSMA-11 PET/CT were read simultaneously by three experienced physicians (at least 5 years of experience in PET reading) searching for mismatch findings. A mismatch finding was defined as metastasis with remarkable [^18^F]FDG uptake and no or considerably less concordant [^68^Ga]Ga-PSMA-11 uptake based on visual analysis. The decision of a mismatch finding was taken in consensus of all PET readers.

### Serum parameters

Selected serum parameters used in our clinical practice were tested as indicators for the occurrence of [^18^F]FDG/[^68^Ga]Ga-PSMA-11 mismatch findings in PET/CT: prostate-specific antigen (PSA), neuron-specific enolase (NSE), gamma-glutamyltransferase (GGT), and alkaline phosphatase (ALP). Absolute values were tested in all 66 patients. Additionally, relative changes (ΔPSA, ΔNSE, ΔGGT, ΔALP) over a period of 4 ± 1 weeks prior to [^18^F]FDG PET/CT were analyzed in 55/66 patients (83.3%). In 11/66 cases (16.7%) no laboratory data from previous blood samples were available.

### Statistical analysis and scoring system

For statistical analysis, Mann-Whitney *U* test was applied using Prism 8 (GraphPad Software, San Diego, USA) to determine significant differences between the groups. A *p* value < 0.05 was regarded as statistically significant. For power calculation of Mann-Whitney *U* test, we calculated the effect size; values higher than 0.5 were considered as a strong effect size. For each parameter that turned out to be statistically significant, a receiver operating characteristic (ROC) analysis was performed and a cut-off point was determined using the maximal value of Youden index. In addition, a scoring system combining all significant parameters was introduced. This scoring system was constructed for ease of clinical use and on the basis of sensitivity-specificity analysis of the strongest parameter, assisted by the other significant parameters, enhancing the power and accuracy of the score.

## Results

**Fig. 1:**
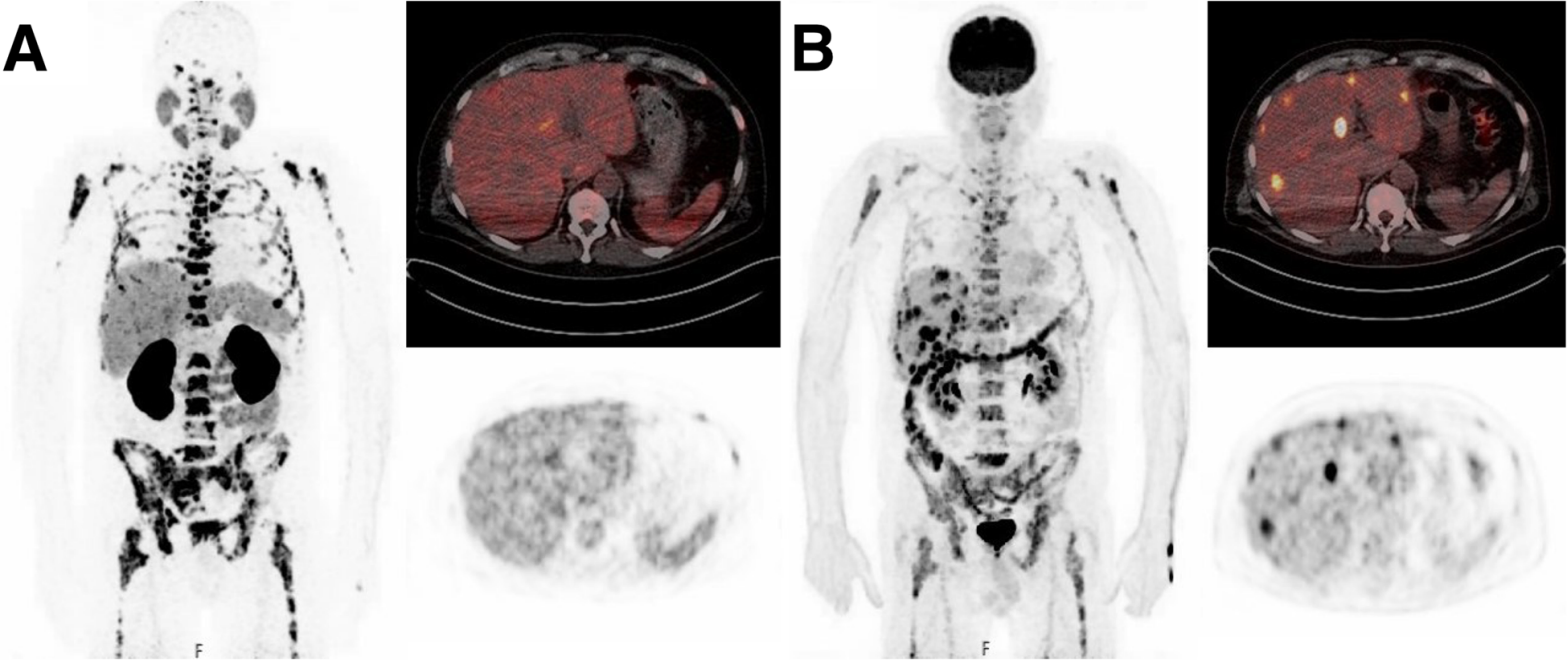
Patient with hepatic mismatch findings. Maximal intensity projection (MIP), PET/CT, and PET data of [^68^Ga]Ga-PSMA-11 PET/CT (**a**) and of [^18^F]FDG PET/CT (**b**)

**Fig. 2:**
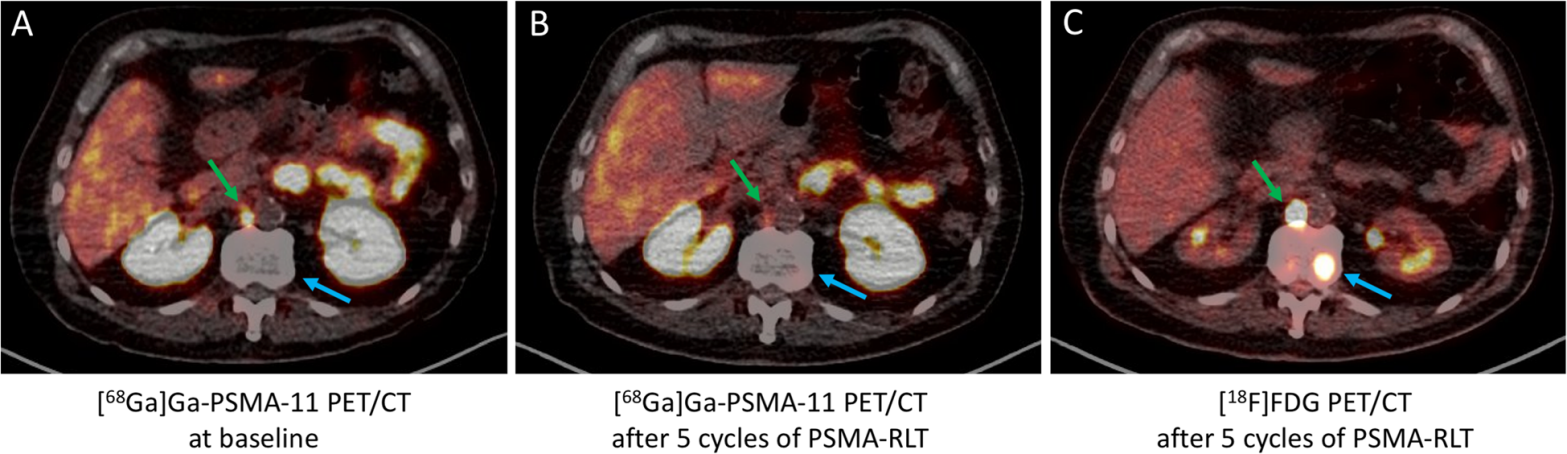
Example of two mismatch lesions detected after 5 cycles of PSMA-RLT: one intensely PSMA-positive (green arrow) and one not identifiable (blue arrow) lesion on the initial [^68^Ga]Ga-PSMA-11 PET/CT at baseline prior to PSMA-RLT (**a**). [^68^Ga]Ga-PSMA-11 PET/CT (**b**) and [^18^F]FDG PET/CT (**c**) after 5 cycles of PSMA-RLT showing [^18^F]FDG/[^68^Ga]Ga-PSMA-11 mismatch in both lesions

In total, 41/66 (62%) patients included in this analysis revealed at least one [^18^F]FDG/[^68^Ga]Ga-PSMA-11 mismatch finding on PET/CT, and 25/66 (38%) had no mismatch findings. Overall, 390 mismatch lesions were detected: 211 in bone (in 24/41 patients), 62 in lymph nodes (in 21/41 patients), 100 in the liver (in 20/41 patients), 4 in the lung (in 1/41 patients), and 13 in other locations (in 9/41 patients). Figure 1 presents an example of a patient with multiple mismatch findings in the liver. In the mismatch group, 13/41 (32%) patients had their mismatch findings (121/390 mismatch lesions) detected at baseline imaging before intended commencement for PSMA-RLT, whereas in the remaining 28/41 (68%) patients the mismatch (269/390 mismatch lesions) was diagnosed in the course of PRLT after having received a mean of 4 ± 2 cycles of PSMA-RLT. When comparing these 269 mismatch lesions to the initial [^68^Ga]Ga-PSMA-11 PET/CT before starting PSMA-RLT, 29/269 (11%) were initially intensely PSMA-positive, 52/269 (19%) were moderately PSMA-positive, and the majority, 188/269 (70%), were not identifiable on initial [^68^Ga]Ga-PSMA-11 PET/CT. Figure 2 shows an example of two mismatch lesions, one intensely PSMA-positive and one not identifiable on the initial [^68^Ga]Ga-PSMA-11 PET/CT.

**Fig. 3:**
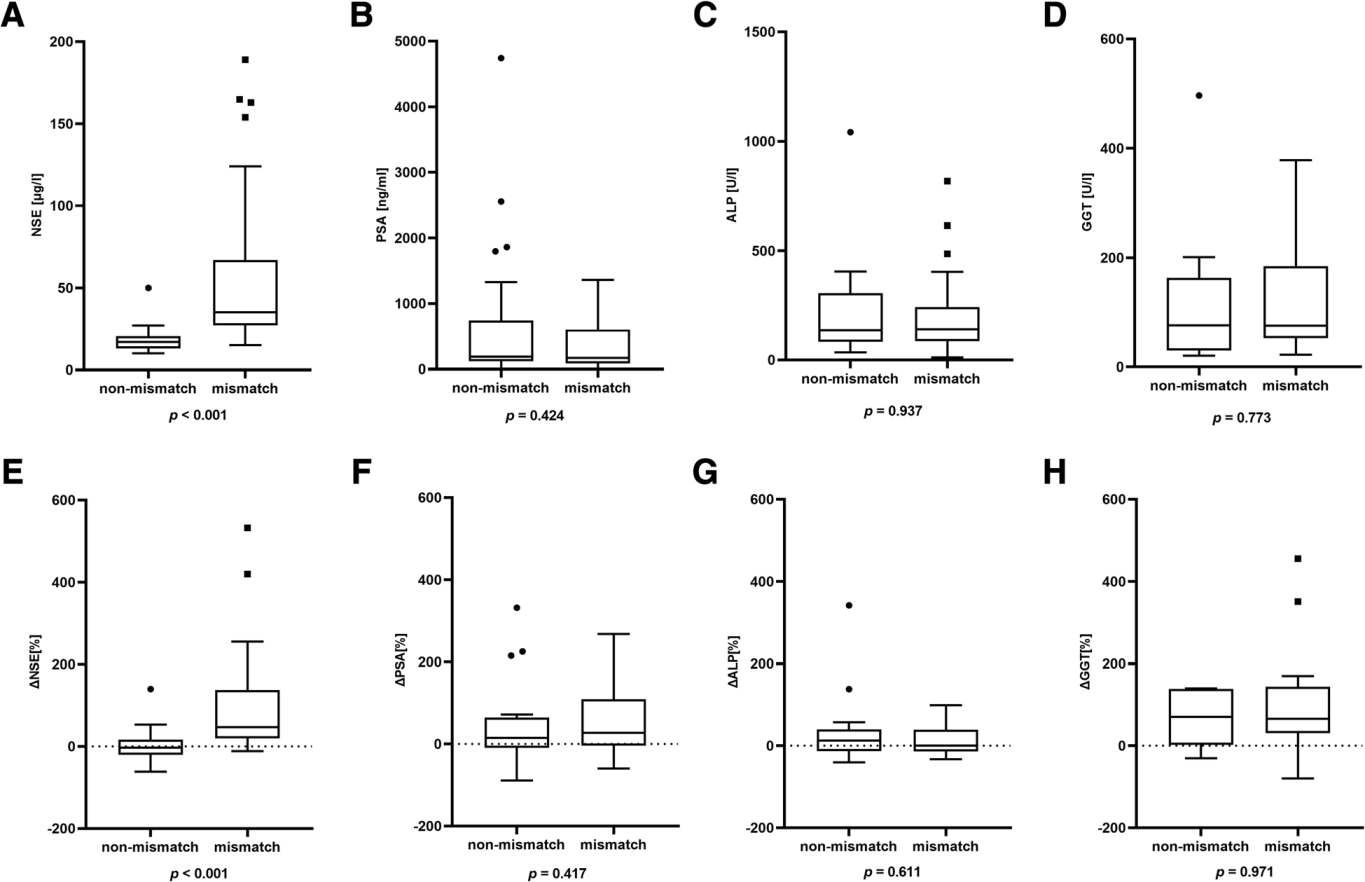
Comparison of absolute serum values of NSE (**a**), PSA (**b**) and ALP (**c**) between all mismatch- and non-mismatch patients. Comparison of serum GGT (**d**) of patients having mismatch and non-mismatch liver metastases. Relative change of each parameter: ΔNSE (**e**), ΔPSA (**f**), ΔALP (**g**), ΔGGT (**h**). Extreme outliers are not shown

**Fig. 4:**
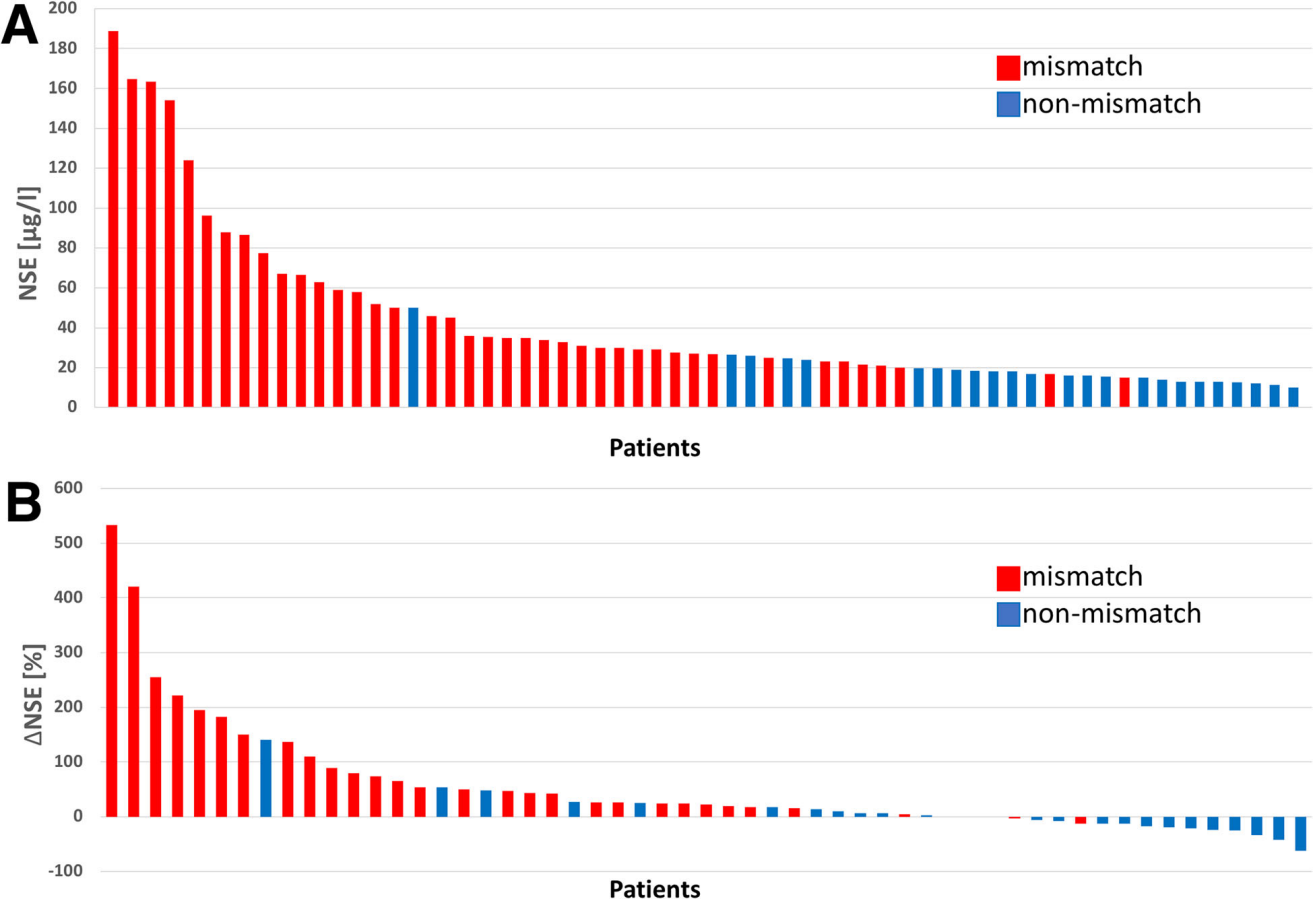
Waterfall plot of NSE (**a**) and ΔNSE (**b**) values in descending order and color coding into mismatch (red) and a non-mismatch (blue)

**Fig. 5:**
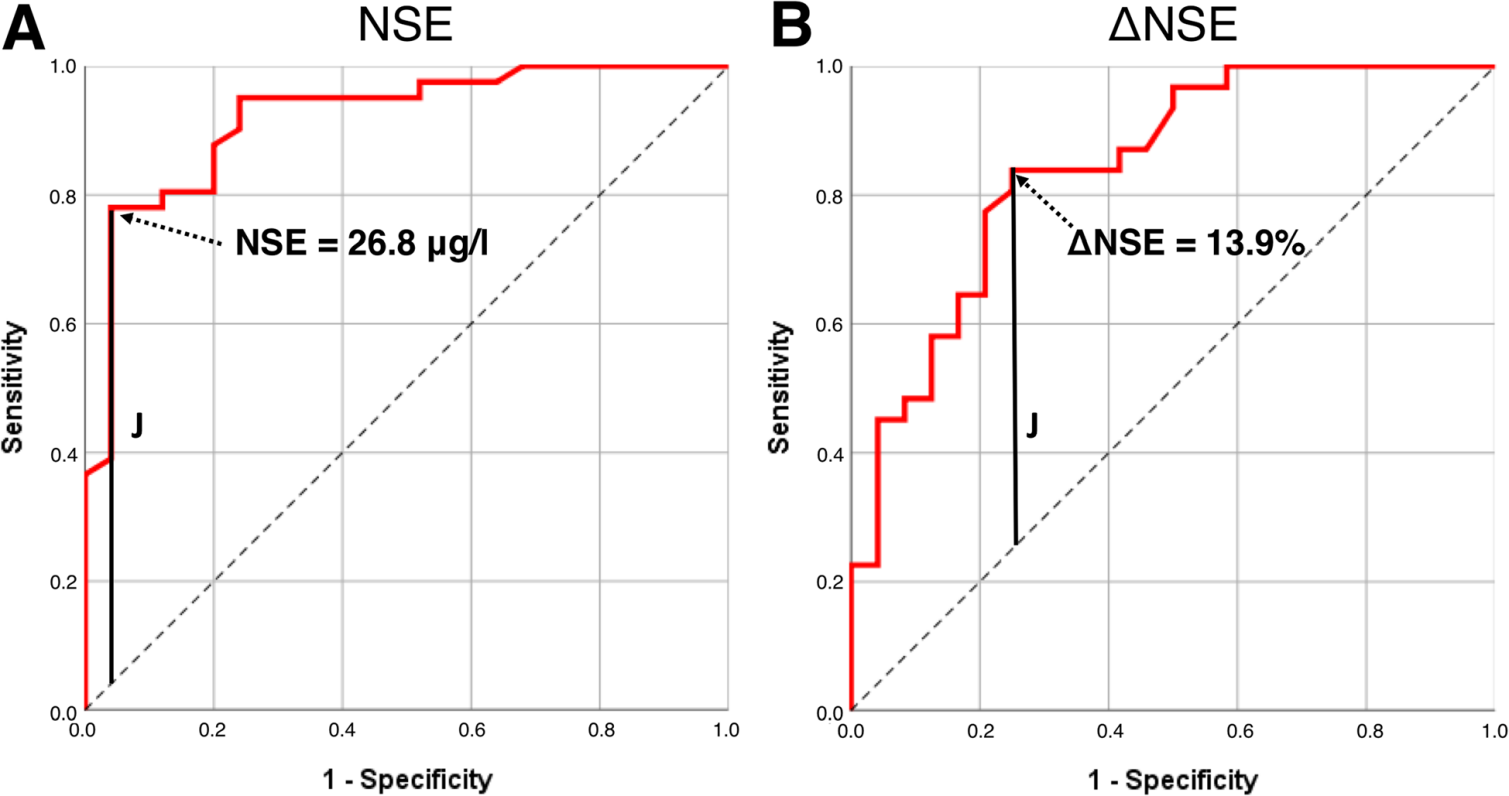
ROC curves for mismatch prediction by serum NSE (**a**) and ΔNSE (**b**) with maximum value of the Youden Index (*J*)

**Fig. 6:**
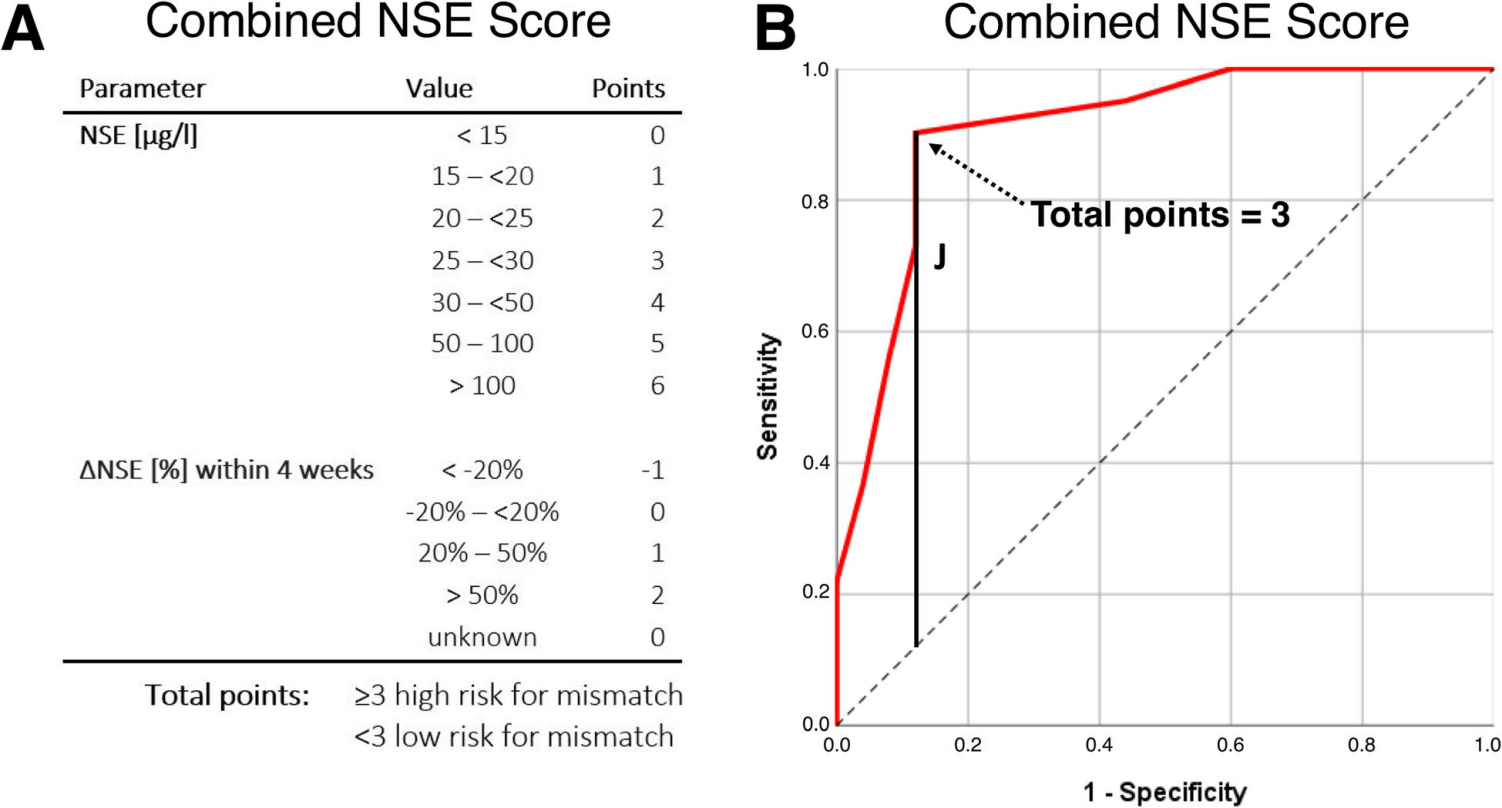
Combined NSE Score (**a**) and ROC curve for mismatch prediction by the score (**b**) with maximum value of the Youden index (*J*)

NSE serum level (55.4 ± 44.6 μg/l vs*.* 18.5 ± 8 μg/l, *p* < 0.001) and ΔNSE (93.8 ± 124.5% vs*.* 2.9 ± 39.5%, *p* < 0.001) were significantly higher in the mismatch group than in the non-mismatch group. No significant difference was found for serum PSA (*p* = 0.424), ΔPSA (*p* = 0.417), serum ALP (*p* = 0.937), and ΔALP (*p* = 0.611) between the mismatch and the non-mismatch group, respectively. Twenty-nine of 66 (44.0%) patients had liver metastases, and 20/29 had at least one mismatch finding in the liver. In patients with liver metastases, GGT (*p* = 0.773) and ΔGGT (*p* = 0.971) also revealed no significant difference between the mismatch and the non-mismatch group. For NSE and ΔNSE, the effect size was 0.70 and 0.59, which were considered as strong. Summarized statistics of the data are presented in Table 2. All these statistical comparisons are illustrated by box-plot diagram format for each tested parameter (Fig. 3). A subgroup analysis comparing the patients diagnosed with a mismatch by screening for PSMA-RLT with the patients diagnosed with a mismatch after several cycles PSMA-RLT revealed no significant difference of serum NSE value (67.5 ± 51.7 μg/l vs*.* 49.9 ± 40.8 μg/l, *p* = 0.12). The predictive impact of NSE and ΔNSE regarding mismatch detection is discernible from the waterfall plots with highlighting of mismatch and non-mismatch individuals (Fig. 4). ROC analyses of NSE and ΔNSE revealed an area under the curve (AUC) of 0.92 and 0.84, respectively (Fig. 5). For NSE, the maximum value of the Youden index (*J* = 0.74) was at a serum level of 26.8 μg/l with a sensitivity of 78% and a specificity of 96% for the occurrence of [^18^F]FDG/[^68^Ga]Ga-PSMA-11 mismatch lesions, whereas the maximum value of the Youden Index for ΔNSE (*J* = 0.59) was at + 13.9% increase with a sensitivity of 84% and a specificity of 75%. Only one patient had mismatch findings with serum NSE < 15 μg/l. On the other hand, we observed several mismatch (13/41) and non-mismatch (16/25) patients with NSE in the range between 15 and 30 μg/l (Fig. 4a). To dichotomize these patients and to improve the sensitivity without marked loss of specificity we introduced a scoring system (Combined NSE Score) based on both parameters, which is presented in Fig. 6a. The main part of the scoring system was assigned to the absolute value of NSE being the strongest parameter in ROC analysis. Zero points were given for NSE in the physiological range of < 15 μg/l. For the ease of clinical use, NSE was classified in the critical range (15–30 μg/l) in steps of 5 μg/l, starting with 1 point for 15–20 μg/l up to 3 points for 25–30 μg/l covering the Youden’s index (26.8 ng/ml) of the sensitivity-specificity analysis. Greater interval steps were chosen for NSE above 30 μg/l (4 points 30–50 μg/l, 5 points 50–100 μg/l and 6 points > 100 μg/l). ΔNSE was additionally included for enhancing the power and accuracy of the score. Additional points for ΔNSE was set to − 1 point when NSE was decreasing (more than − 20 %), to 0 points with stable (− 20 to + 20 %) or unknown NSE, and to 1 (20–50%) or to 2 (> 50%) points for increasing NSE. The ROC analysis of the developed scoring system (Fig. 6b) revealed an AUC of 0.91 and a maximum value of the Youden index of 0.78 at 3 scoring points with a sensitivity of 90% and a specificity of 88% for the occurrence of [^18^F]FDG/[^68^Ga]Ga-PSMA-11 mismatch lesions. The gain of the Combined NSE Score is seen at the sensitivity level of about 90%, where the isolated use of NSE achieves only a specificity of 76%.

**Table 2:** Descriptive statistics of serum parameters

	Group	***n***	Median (IQR)	Mean (± SD)	Minimum	Maximum	***p*** value
**NSE [μg/l]**	Mismatch	41	35 (30.0–66.9)	55.4 (± 44.6)	15	188.6	**< 0.001**
	Non-mismatch	25	16.9 (13.0–20.4)	18.5 (± 8.0)	10	50	
**PSA [ng/ml]**	Mismatch	41	168 (83.0–602.5)	367.6 (± 407.9)	0.3	1360	0.424
	Non-mismatch	25	190 (116.8–743.0)	666.3 (± 1086.8)	3	4742	
**GGT [U/l]**	Mismatch	22	75 (52.5–184.5)	120.5 (± 104.5)	22	378	0.773
	Non-mismatch	9	76 (30.0–163.0)	127 (± 149.9)	20	497	
**ALP [U/l]**	Mismatch	41	140 (86–242.5)	192.2 (± 163.5)	12	818	0.937
	Non-mismatch	25	136 (83.5–306.5)	211.8 (± 209.6)	35	1042	
**ΔNSE [%]**	Mismatch	31	47 (18.6–137.3)	93.8 (± 124.5)	− 12	533	**< 0.001**
	Non-mismatch	24	3 (− 20.5 to 15.6)	2.9 (± 39.5)	− 62	140	
**ΔPSA [%]**	Mismatch	31	27 (− 4.3 to 109.1)	254.7 (± 890.3)	− 60	4956	0.417
	Non-mismatch	24	15 (− 9.7 to 64.4)	63.8 (± 153.5)	− 89	631	
**ΔGGT [%]**	Mismatch	18	65.5 (30.3–143.8)	196.8 (± 464.9)	− 79	1986	0.971
	Non-mismatch	9	70 (2.0–138.5)	205.3 (± 454.6)	− 31	1406	
**ΔALP [%]**	Mismatch	31	0 (− 14.0 to 39.0)	35.3 (± 137.6)	− 33	753	0.611
	Non-mismatch	24	12.5 (− 13.8 to 40.0)	28.2 (± 76.4)	− 41	342	

*Abbreviations*: *NSE* neuron-specific enolase, *PSA* prostate-specific antigen, *GGT* gamma-glutamyltransferase, *ALP* alkaline phosphatase

## Discussion

We observed a significantly higher absolute serum concentration and a higher relative increase of NSE in advanced mCRPC patients with [^18^F]FDG-avid and insufficient PSMA expressing metastases ([^18^F]FDG/[^68^Ga]Ga-PSMA-11 mismatch findings) in our cohort.

NSE is a highly specific marker for neurons and peripheral neuroendocrine cells, used as a biomarker for aggressive forms of neuroendocrine tumors and small cell carcinoma [27, 28]. Elevated NSE serum levels have also been observed in neuroendocrine subtypes of prostate carcinoma [29, 30]. While only a small proportion of prostate cancer patients represent neuroendocrine features at the beginning of disease [31], more patients develop these under therapy [24]. Serum NSE might be increased in patients with mismatch findings as a sign of dedifferentiation or transformation to a neuroendocrine subtype. This could be a mechanism of resistance induced by selective treatment pressure and was particularly observed in patients undergoing anti-androgen therapy [32–34]. Neuroendocrine differentiation in prostate carcinoma is a phenotypic change by which prostate cancer cells trans-differentiate into neuroendocrine-like cells. These neuroendocrine-like cells are lacking expression of androgen receptor and prostate-specific antigen. Neuroendocrine-like cells are known to produce peptide hormones and growth factors to promote tumor progression and are apoptosis-resistant contributing to treatment failure [35–37]. Some case reports described that PSMA-targeted PET/CT may be not able to detect neuroendocrine prostate carcinoma [38–40]. Supporting these observations, Bakht et al. (2018) demonstrated in vitro on patient-derived xenograft (PDX) an inverse correlation between PSMA gene (FOLH1) and neuroendocrine biomarker gene expression [41]. Particularly, suppression of the PSMA gene was observed in 65% of cases which overexpressed the NSE gene (ENO2). They also demonstrated that the most progressions’ pathway to neuroendocrine trans-differentiation under ongoing treatment was associated subsequently with loss of the PSMA expression. Thus, alternative molecular imaging methods are required for neuroendocrine phenotype.

The glucose transporter GLUT1 has been found to be overexpressed on cells of the advanced late-stage prostate cancer and its expression was related to tumor aggressiveness [42]. Meziou et al. (2020) assumed that GLUT1 expression may be increased in neuroendocrine prostate carcinoma suggesting that [^18^F]FDG PET/CT might be an important imaging tool to examine neuroendocrine prostate carcinoma [43]. Few studies demonstrated the clinical utility of [^18^F]FDG PET/CT in neuroendocrine prostate carcinoma [44–46]. Spratt et al. demonstrated in 23 patients with neuroendocrine prostate cancer that [^18^F]FDG PET/CT has clinical benefit and reported a high detection rate of metastatic disease, especially of lymph node and visceral metastases [46].

This hypothesis of transformation to prostate cancer with neuroendocrine features in our mismatch cohort was confirmed histopathologically in one patient only. More evidence is required to confirm this hypothesis. Thus, consistent histopathological examination of mismatch metastases should be performed in future studies.

Patients with mCRPC, presenting discordant [^18^F]FDG-avid lesions with low or no PSMA expression, have a poor prognosis with very short survival time [47]. This mismatch phenomenon indicates a more aggressive type of mCRPC in clinical routine and precludes patients from PSMA-directed radioligand therapy [9]. Thus, early diagnosis of mismatch findings is very important before and during PSMA-RLT to provide these patients with alternative therapy options in addition to or instead of PSMA-RLT, including chemotherapy, immunotherapy, bone-seeking radiopharmaceuticals, PARP inhibition, and other novel targeted treatments [18, 47].

Our results obtained from 66 mCRPC patients suggest that serum NSE is a potential biomarker for the existence of the described [^18^F]FDG/[^68^Ga]Ga-PSMA-11 mismatch. Statistical analysis of our data proposed an NSE cut-off value for a mismatch occurrence of 26.8 μg/l (sensitivity 78%, specificity 96%) and of + 13.9 % increase for ΔNSE (sensitivity 84%, specificity 75%). A combination of both parameters may improve the predictive power. We achieved a sensitivity and specificity of almost 90% with our introduced Combined NSE Score (cut-off: total points ≥ 3, Fig. 6). The simplicity of the score allows easy clinical application as a guide for further diagnostic procedures in the mCRPC setting.

In contrast to NSE, other serum parameters analyzed in this study as PSA, ALP, and GGT were not able to indicate mismatch findings in our cohort. These biomarkers may only reflect total tumor burden giving no specific hint towards [^18^F]FDG-avid lesions with low or missing PSMA expression.

We suggest including NSE assessments into routine blood tests of mCRPC patients before and during PSMA-RLT. However, these results should be noticed with caution considering a potential bias due to the retrospective study design and not-representative patient cohort. Quite often, [^18^F]FDG PET/CT was performed when viable PSMA-negative metastases were suspected before or during PSMA-RLT due to a worsening course of disease or by hint in other imaging methods (CT, MRI). Consequently, more mismatch patients (62% in our cohort) than non-mismatch patients were included in our study, resulting in a preselected cohort of mCRPC patients not representing the normal incidence of mismatch in candidates referred for PSMA-RLT. In a prospective phase-II trial for PSMA-RLT, Hofman et al. (2019) reported an incidence of 16.3% (*n* = 7/43) for [^18^F]FDG/[^68^Ga]Ga-PSMA-11 mismatch at time of screening for PSMA-RLT [20]. This was the first prospective study which excluded patients with mismatch lesions. A prospective setting including larger non-biased patient cohorts is necessary to confirm our findings. Further limitations are the missing blood samples in 11/66 (16.7%) patients prior to screening. Those patients were included in the scoring system as “unknown” ΔNSE, which reflects a frequent clinical situation in mCRPC patients referred for evaluation of PSMA-RLT. As another point of criticism, no quantitative SUV threshold defining low PSMA expression on [^68^Ga]Ga-PSMA-11 PET/CT was set, which is also a global problem in literature suffering from defined cut-offs for adequate PSMA expression. Furthermore, additional serum parameters, which were recently reported as prognostic markers in the mCRPC setting for PSMA-RLT, such as the neuroendocrine marker chromogranin A or the lactate dehydrogenase (LDH) [48–50], were not available for testing in this study. Serum chromogranin A values might also be higher in patients with mismatch findings similar to NSE. LDH, which is a marker with strong prognostic value for response prediction of PSMA-RLT [48], might also be a potential marker for mismatch. Both parameters should thus also be tested in future studies. If those studies would indicate a mismatch predictive value, these parameters could be included to the scoring system to increase its sensitivity and specificity. Lastly, histopathological confirmation of a transformation process in mismatch lesions towards a neuroendocrine phenotype was performed only in one case. Histopathological examination of mismatch metastases should be included in future prospective studies.

## Conclusions

We observed a significantly higher absolute serum concentration and a higher relative increase of NSE in advanced mCRPC patients with [^18^F]FDG-avid and insufficient PSMA expressing metastases ([^18^F]FDG/[^68^Ga]Ga-PSMA-11 mismatch findings on PET/CT) in our cohort. NSE might be used as a potential laboratory indicator for [^18^F]FDG/[^68^Ga]Ga-PSMA-11 mismatch findings, if this observation is confirmed in future, ideally prospective, studies in larger patient cohorts.

## Data Availability

The datasets used and analyzed during the current study are available from the corresponding author on reasonable request.

## Abbreviations

ADT: Androgen deprivation therapy
ALP: Alkaline phosphatase
CI: 95% confidence interval of the mean
CT: Computer tomography
FDG: Fluorodeoxyglucose
GGT: Gamma-glutamyltransferase
LDH: Lactate dehydrogenase
mCRPC: Metastasized castration-resistant prostate carcinoma
NSE: Neuron-specific enolase
PET: Positron emission tomography
PSA: Prostate-specific antigen
PSMA: Prostate-specific membrane antigen
PSMA-RLT: PSMA-targeted radioligand therapy
ROC: Receiver operating characteristic

## References

[1] Bray F, Ferlay J, Soerjomataram I, Siegel RL, Torre LA, Jemal A (2018). Global cancer statistics 2018: GLOBOCAN estimates of incidence and mortality worldwide for 36 cancers in 185 countries. CA Cancer J Clin..

[2] Kirby M, Hirst C, Crawford ED (2011). Characterising the castration-resistant prostate cancer population: a systematic review: The Epidemiology of CRPC. Int J Clin Pract..

[3] Watson PA, Arora VK, Sawyers CL (2015). Emerging mechanisms of resistance to androgen receptor inhibitors in prostate cancer. Nat Rev Cancer..

[4] Cornford P, Bellmunt J, Bolla M, Briers E, De Santis M, Gross T (2017). EAU-ESTRO-SIOG Guidelines on Prostate Cancer. Part II: treatment of relapsing, metastatic, and castration-resistant prostate cancer. Eur Urol..

[5] Berthold DR, Pond GR, Soban F, de Wit R, Eisenberger M, Tannock IF (2008). Docetaxel plus prednisone or mitoxantrone plus prednisone for advanced prostate cancer: Updated Survival in the TAX 327 Study. J Clin Oncol..

[6] Tannock IF, Horti J, Oudard S, James ND, Rosenthal MA (2004). Docetaxel plus prednisone or mitoxantrone plus prednisone for advanced prostate cancer. N Engl J Med..

[7] Ryan CJ, Smith MR, de Bono JS, Molina A, Logothetis CJ, de Souza P (2013). Abiraterone in metastatic prostate cancer without previous chemotherapy. N Engl J Med..

[8] Beer TM, Armstrong AJ, Rathkopf DE, Loriot Y, Sternberg CN, Higano CS (2014). Enzalutamide in metastatic prostate cancer before chemotherapy. N Engl J Med..

[9] Kratochwil C, Fendler WP, Eiber M, Baum R, Bozkurt MF, Czernin J (2019). EANM procedure guidelines for radionuclide therapy with ^177^Lu-labelled PSMA-ligands (^177^Lu-PSMA-RLT). Eur J Nucl Med Mol Imaging..

[10] Yadav MP, Ballal S, Tripathi M, Damle NA, Sahoo RK, Seth A (2017). ^177^Lu-DKFZ-PSMA-617 therapy in metastatic castration resistant prostate cancer: safety, efficacy, and quality of life assessment. Eur J Nucl Med Mol Imaging..

[11] Fendler WP, Rahbar K, Herrmann K, Kratochwil C, Eiber M (2017). ^177^Lu-PSMA radioligand therapy for prostate cancer. J Nucl Med..

[12] Kim YJ, Kim Y (2018). Therapeutic responses and survival effects of ^177^Lu-PSMA-617 radioligand therapy in metastatic castrate-resistant prostate cancer: a meta-analysis. Clin Nucl Med..

[13] Afshar-Oromieh A, Babich JW, Kratochwil C, Giesel FL, Eisenhut M, Kopka K (2016). The rise of PSMA ligands for diagnosis and therapy of prostate cancer. J Nucl Med..

[14] Heinzel A, Boghos D, Mottaghy FM, Gaertner F, Essler M, von Mallek D (2019). ^68^Ga-PSMA PET/CT for monitoring response to ^177^Lu-PSMA-617 radioligand therapy in patients with metastatic castration-resistant prostate cancer. Eur J Nucl Med Mol Imaging..

[15] Kuten J, Sarid D, Yossepowitch O, Mabjeesh NJ, Even-Sapir E. [^68^Ga]Ga-PSMA-11 PET/CT for monitoring response to treatment in metastatic prostate cancer: is there any added value over standard follow-up? EJNMMI Res. 2019;9:84. 10.1186/s13550-019-0554-1 PMC6715755 31468235

[16] Rahbar K, Afshar-Oromieh A, Jadvar H, Ahmadzadehfar H (2018). PSMA Theranostics: current status and future directions. Mol Imaging..

[17] Alipour R, Azad A, Hofman MS (2019). Guiding management of therapy in prostate cancer: time to switch from conventional imaging to PSMA PET?. Ther Adv Med Oncol..

[18] Emmett L, Crumbaker M, Ho B, Willowson K, Eu P, Ratnayake L (2019). Results of a prospective phase 2 pilot trial of ^177^Lu–PSMA-617 therapy for metastatic castration-resistant prostate cancer including imaging predictors of treatment response and patterns of progression. Clin Genitourin Cancer..

[19] Eidelman E, Twum-Ampofo J, Ansari J, Siddiqui MM (2017). The metabolic phenotype of prostate cancer. Front Oncol..

[20] Hofman MS, Violet J, Hicks RJ, Ferdinandus J, Thang SP, Akhurst T (2018). [^177^Lu]-PSMA-617 radionuclide treatment in patients with metastatic castration-resistant prostate cancer (LuPSMA trial): a single-centre, single-arm, phase 2 study. Lancet Oncol..

[21] Petrylak DP, Crawford ED (2017). Biomarkers for the Management of Castration-Resistant Prostate Cancer: We Are Not There Yet. Target Oncol..

[22] Heinrich D, Bruland Ø, Guise TA, Suzuki H, Sartor O (2018). Alkaline phosphatase in metastatic castration-resistant prostate cancer: reassessment of an older biomarker. Future Oncol..

[23] Whitfield JB (2001). Gamma glutamyl transferase. Crit Rev Clin Lab Sci..

[24] Grigore AD, Ben-Jacob E, Farach-Carson MC (2015). Prostate cancer and neuroendocrine differentiation: more neuronal, less endocrine?. Front Oncol..

[25] Fendler Wolfgang P., Eiber Matthias, Beheshti Mohsen, Bomanji Jamshed, Ceci Francesco, Cho Steven, Giesel Frederik, Haberkorn Uwe, Hope Thomas A., Kopka Klaus, Krause Bernd J., Mottaghy Felix M., Schöder Heiko, Sunderland John, Wan Simon, Wester Hans-Jürgen, Fanti Stefano, Herrmann Ken (2017). 68Ga-PSMA PET/CT: Joint EANM and SNMMI procedure guideline for prostate cancer imaging: version 1.0. European Journal of Nuclear Medicine and Molecular Imaging.

[26] Krause BJ, Beyer T, Bockisch A, Delbeke D, Kotzerke J, Minkov V (2007). FDG-PET/CT in oncology. Nuklearmedizin. Schattauer GmbH.

[27] Schmechel D, Marangos PJ, Brightman M (1978). Neurone-specific enolase is a molecular marker for peripheral and central neuroendocrine cells. Nature..

[28] Isgrò MA, Bottoni P, Scatena R (2015). Neuron-specific enolase as a biomarker: biochemical and clinical aspects. Adv Exp Med Biol..

[29] Terry S, Beltran H (2014). The many faces of neuroendocrine differentiation in prostate cancer progression. Front Oncol..

[30] Muoio B, Pascale M, Roggero E (2018). The role of serum neuron-specific enolase in patients with prostate cancer: a systematic review of the recent literature. Int J Biol Markers..

[31] Marcus DM, Goodman M, Jani AB, Osunkoya AO, Rossi PJ (2012). A comprehensive review of incidence and survival in patients with rare histological variants of prostate cancer in the United States from 1973 to 2008. Prostate Cancer Prostatic Dis..

[32] Beltran H, Tomlins S, Aparicio A, Arora V, Rickman D, Ayala G (2014). Aggressive variants of castration resistant prostate cancer. Clin Cancer Res..

[33] Aggarwal R, Zhang T, Small EJ, Armstrong AJ (2014). Neuroendocrine prostate cancer: subtypes, biology, and clinical outcomes. J Natl Compr Canc Netw..

[34] Ismail AHR, Landry F, Aprikian AG, Chevalier S (2002). Androgen ablation promotes neuroendocrine cell differentiation in dog and human prostate. Prostate..

[35] Hu C-D (2015). Choo R. Huang J. Neuroendocrine differentiation in prostate cancer: a mechanism of radioresistance and treatment failure. Front Oncol..

[36] Xing N, Qian J, Bostwick D, Bergstralh E, Young CY (2001). Neuroendocrine cells in human prostate over-express the anti-apoptosis protein survivin. Prostate..

[37] Heinrich E, Probst K, Michel MS, Trojan L (2011). Gastrin-releasing peptide: predictor of castration-resistant prostate cancer?. The Prostate..

[38] Usmani S, Ahmed N, Marafi F, Rasheed R, Amanguno HG, Al KF (2017). Molecular imaging in neuroendocrine differentiation of prostate cancer: 68Ga-PSMA versus ^68^Ga-DOTA NOC PET-CT. Clin Nucl Med..

[39] Chakraborty PS, Tripathi M, Agarwal KK, Kumar R, Vijay MK, Bal C (2015). Metastatic poorly differentiated prostatic carcinoma with neuroendocrine differentiation: negative on ^68^Ga-PSMA PET/CT. Clin Nucl Med..

[40] Tosoian JJ, Gorin MA, Rowe SP, Andreas D, Szabo Z, Pienta KJ (2017). Correlation of PSMA-targeted ^18^F-DCFPyL PET/CT findings with immunohistochemical and genomic data in a patient with metastatic neuroendocrine prostate cancer. Clin Genitourin Cancer..

[41] Bakht MK, Derecichei I, Li Y, Ferraiuolo R-M, Dunning M, Oh SW (2018). Neuroendocrine differentiation of prostate cancer leads to PSMA suppression. Endocr Relat Cancer..

[42] Stewart GD, Gray K, Pennington CJ, Edwards DR, Riddick ACP, Ross JA (2008). Analysis of hypoxia-associated gene expression in prostate cancer: lysyl oxidase and glucose transporter-1 expression correlate with Gleason score. Oncol Rep..

[43] Meziou S, Ringuette Goulet C, Hovington H, Lefebvre V, Lavallée É, Bergeron M, et al. GLUT1 expression in high-risk prostate cancer: correlation with ^18^F-FDG-PET/CT and clinical outcome. Prostate Cancer Prostatic Dis. 2020. 10.1038/s41391-020-0202-x 31932660

[44] Liu Y (2008). FDG PET-CT demonstration of metastatic neuroendocrine tumor of prostate. World J Surg Oncol..

[45] Parida GK, Tripathy S, Datta Gupta S, Singhal A, Kumar R, Bal C (2018). Adenocarcinoma prostate with neuroendocrine differentiation: potential utility of ^18^F-FDG PET/CT and ^68^Ga-DOTANOC PET/CT over ^68^Ga-PSMA PET/CT. Clin Nucl Med..

[46] Spratt DE, Gavane S, Tarlinton L, Fareedy SB, Doran MG, Zelefsky MJ (2014). Utility of FDG-PET in clinical neuroendocrine prostate cancer. Prostate..

[47] Thang SP, Violet J, Sandhu S, Iravani A, Akhurst T, Kong G (2019). Poor outcomes for patients with metastatic castration-resistant prostate cancer with low prostate-specific membrane antigen (PSMA) expression deemed ineligible for ^177^Lu-labelled PSMA radioligand therapy. Eur Urol Oncol..

[48] Rathke H, Holland-Letz T, Mier W, Flechsig P, Mavriopoulou E, Röhrich M, et al. Response prediction of ^177^Lu-PSMA-617 RLT using PSA, Chromogranin A, and LDH. J Nucl Med. 2019;jnumed.119.231431. 10.2967/jnumed.119.231431 31653712

[49] Yordanova A, Linden P, Hauser S, Feldmann G, Brossart P, Fimmers R (2020). The value of tumor markers in men with metastatic prostate cancer undergoing [^177^Lu]Lu-PSMA therapy. The Prostate..

[50] Heck MM, Tauber R, Schwaiger S, Retz M, D’Alessandria C, Maurer T (2019). Treatment outcome, toxicity, and predictive factors for radioligand therapy with ^177^Lu-PSMA-I&T in metastatic castration-resistant prostate cancer. European Urology..

